# 2′,3′-cAMP treatment mimics the stress molecular response in *Arabidopsis thaliana*

**DOI:** 10.1093/plphys/kiac013

**Published:** 2022-01-19

**Authors:** Monika Chodasiewicz, Olga Kerber, Michal Gorka, Juan C Moreno, Israel Maruri-Lopez, Romina I Minen, Arun Sampathkumar, Andrew D L Nelson, Aleksandra Skirycz

**Affiliations:** Center for Desert Agriculture, Biological and Environmental Science and Engineering Division (BESE), King Abdullah University of Science and Technology (KAUST), Thuwal, Saudi Arabia; Max Planck Institute of Molecular Plant Physiology, 14476 Potsdam, Germany; Max Planck Institute of Molecular Plant Physiology, 14476 Potsdam, Germany; Max Planck Institute of Molecular Plant Physiology, 14476 Potsdam, Germany; Center for Desert Agriculture, Biological and Environmental Science and Engineering Division (BESE), King Abdullah University of Science and Technology (KAUST), Thuwal, Saudi Arabia; Max Planck Institute of Molecular Plant Physiology, 14476 Potsdam, Germany; Center for Desert Agriculture, Biological and Environmental Science and Engineering Division (BESE), King Abdullah University of Science and Technology (KAUST), Thuwal, Saudi Arabia; Boyce Thompson Institute, Cornell University, Ithaca, New York 14853, USA; Max Planck Institute of Molecular Plant Physiology, 14476 Potsdam, Germany; Boyce Thompson Institute, Cornell University, Ithaca, New York 14853, USA; Max Planck Institute of Molecular Plant Physiology, 14476 Potsdam, Germany; Boyce Thompson Institute, Cornell University, Ithaca, New York 14853, USA

## Abstract

The role of the RNA degradation product 2′,3′-cyclic adenosine monophosphate (2′,3′-cAMP) is poorly understood. Recent studies have identified 2′,3′-cAMP in plant material and determined its role in stress signaling. The level of 2′,3′-cAMP increases upon wounding, in the dark, and under heat, and 2′,3′-cAMP binding to an RNA-binding protein, Rbp47b, promotes stress granule (SG) assembly. To gain further mechanistic insights into the function of 2′,3′-cAMP, we used a multi-omics approach by combining transcriptomics, metabolomics, and proteomics to dissect the response of Arabidopsis (*Arabidopsis thaliana*) to 2′,3′-cAMP treatment. We demonstrated that 2′,3′-cAMP is metabolized into adenosine, suggesting that the well-known cyclic nucleotide–adenosine pathway of human cells might also exist in plants. Transcriptomics analysis revealed only minor overlap between 2′,3′-cAMP- and adenosine-treated plants, suggesting that these molecules act through independent mechanisms. Treatment with 2′,3′-cAMP changed the levels of hundreds of transcripts, proteins, and metabolites, many previously associated with plant stress responses, including protein and RNA degradation products, glucosinolates, chaperones, and SG components. Finally, we demonstrated that 2′,3′-cAMP treatment influences the movement of processing bodies, confirming the role of 2′,3′-cAMP in the formation and motility of membraneless organelles.

## Teaser

2′,3′-cAMP induces changes in plant cells that correspond to changes induced by stress.

## Introduction

To cope with a fluctuating environment, living organisms developed signaling mechanisms to rapidly respond and acclimate to changing conditions. Signaling cascades comprise diverse protein and small molecule players, which act through a series of timely and spatially spaced interactions to regulate the activity, localization, and aggregation of downstream targets driving physiological alterations (Catozzi et al., 2016). Cyclic nucleotides are a group of important and evolutionarily conserved signaling small molecules (Manganiello and Degerman, 1999). In human cells, 3′,5′-cyclic adenosine monophosphate (cAMP) acts as a second messenger downstream of adrenaline and glucagon but upstream of sugar and lipid metabolism. In contrast with 3′,5′-cAMP, its positional isomer—2′,3′-cAMP—has received considerably less research attention. In fact, 2′,3′-cAMP was only discovered in 2009 in a biological material (Ren et al., 2009). Further functional studies showed that 2′,3′-cAMP is a product of 3′→5′ RNA degradation (Thompson et al., 1994) and thus accumulates under conditions characterized by excessive mRNA decay, such as tissue injury (Jackson et al., 2009; Verrier et al., 2012; Van Damme et al., 2014). Although this attribute is also shared by other 2’,3’-cyclic nucleotide monophospates (2′,3′-cNMPs), 2′,3′-cAMP is the most abundant, mainly owing to the presence of the mRNA poly(A) tail. In animal cells, high levels of cellular 2′,3′-cAMP are considered toxic and have been linked to mitochondrial dysfunction (Azarashvili et al., 2009). Furthermore, animal cells can efficiently metabolize 2′,3′-cAMP, first to 2′-AMP via the activity of the 2′,3′-cyclic nucleotide-3′-phosphodiesterase and subsequently to adenosine (Jackson et al., 2009; Jackson, 2016). Given that adenosine exhibits health-promoting properties (Jackson et al., 2009), the conversion of 2′,3′-cAMP to adenosine is proposed as a switch from a toxic to a non-toxic cellular environment.

In both animal and plant cells, cellular levels of 2′,3′-cAMP increase under stress treatments, such as wounding (Van Damme et al., 2014) or heat and dark conditions (Kosmacz et al., 2018). Moreover, 2′,3′-cAMP interacts with RNA-binding protein 47b (Rbp47b) (Kosmacz et al., 2018; Kosmacz and Skirycz, 2020), an important protein for stress granule (SG) formation (Kosmacz et al., 2019). These findings support the role of 2′,3′-cAMP in stress signaling and regulation. SGs are membraneless organelles formed in response to stress (Sorenson and Bailey-Serres, 2014; Gutierrez-Beltran et al., 2015; Jang et al., 2020). In addition to the core proteins required for SG assembly and maintenance, such as protein and RNA chaperones, SGs sequester various metabolic enzymes and regulators. SGs are tightly linked to a different membraneless organelle called processing bodies (PBs) (Kedersha et al., 2005), and both are involved in the regulation of the fate of mRNA; they affect the storage, degradation, and translation of mRNA (Chantarachot and Bailey-Serres, 2018).

To gain further mechanistic insights into the function of 2′,3′-cAMP, we used transcriptomics, metabolomics, and proteomics to dissect the Arabidopsis response to supplementation with a permeable analog of 2′,3′-cAMP, Br-2′,3′-cAMP. We used the Br analog because without this modification, cyclic nucleotides cannot efficiently enter cells (Robison et al., 1965). The Br derivatives of both 3′,5′-cAMP and 2′,3′-cAMP have been successfully used to study cAMP signaling and regulation in plants (Alqurashi et al., 2016; Kosmacz et al., 2018) and animals (Huising et al., 2007). Br-3′,5′-cAMP activates protein kinase A as efficiently as 3′,5′-cAMP, demonstrating that the addition of a bromide group does not interfere with cAMP physiological activity (Yao et al., 2015). Similarly, both exogenously supplied 2′,3′-cAMP and Br-2′,3′-cAMP induce exosome production in human carcinoma cells; however, the effective concentration of Br-2′,3′-cAMP is 10-fold lower than that of 2′,3′-cAMP, which is attributed to the difference in their uptake (Ludwig et al., 2020). Thus, we have evaluated the Arabidopsis response to Br-2′,3′-cAMP treatment at the molecular level by combining complementary transcriptomics, proteomics, and metabolomics experiments. Our data revealed the following: (1) Br-2′,3′-cAMP is taken up by plants, where it is metabolized to Br-adenosine; (2) Br-2′,3′-cAMP treatment triggers major responses at the transcript, protein, and metabolite levels, bearing a known stress signature; and (3) Br-2′,3′-cAMP treatment affects the abundance of key SG proteins and induces PB movement.

## Results

### Treatment with 2′,3′-cAMP leads to the accumulation of stress-responsive metabolites

**Figure 1:**
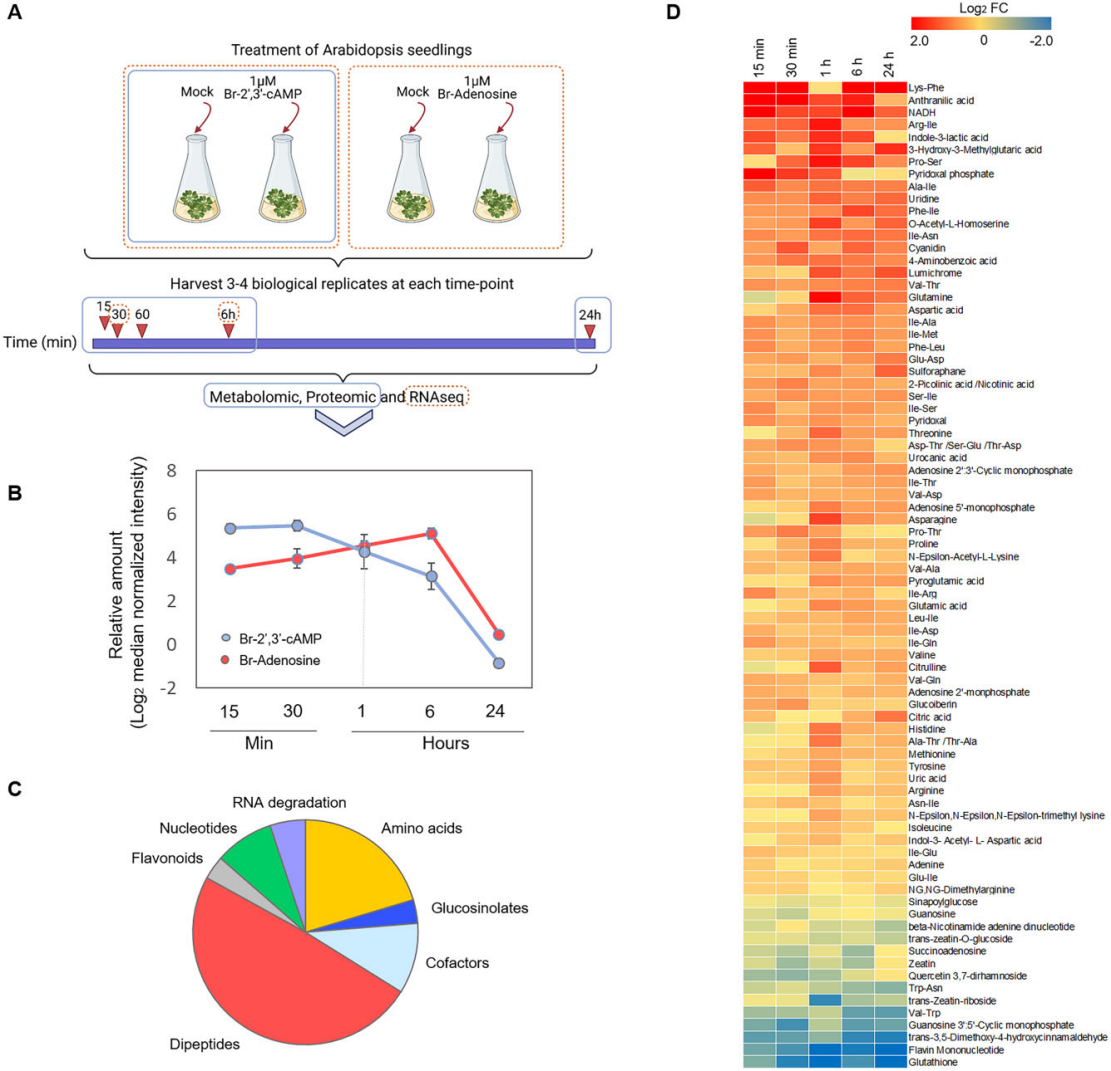
Br-2′,3′-cAMP treatment induces stress-responsive changes at the metabolome level. A, Schematic of the experimental design. Arabidopsis wild-type plants were treated with mock, 1-μM Br-2′,3′-cAMP, or 1-µM Br-adenosine (for RNAseq analysis: orange rectangle/dashed lines). A total of three to four biological samples were collected at five time points for proteomics and metabolomics analyses (blue rectangle)—15 min, 30 min, 1 h, 6 h, and 24 h—and at only two time points for transcriptome analysis (orange dashed line/circles)—15 min and 6 h. Samples were extracted and prepared for proteomics, metabolomics, and RNAseq analyses. Data were analyzed with a focus on significant 2′,3′-cAMP-induced changes. B, Change in the levels of Br-2′,3′-cAMP and Br-adenosine in plants treated with 1 µM Br-2′,3′-cAMP. Data are presented as mean of log2 median normalized intensity. Error bars represent standard deviation, *n* = 4. C, The groups of metabolites which level significantly changed upon 2′,3′-cAMP treatment (two-way ANOVA, *P*-value FDR corrected  ≤ 0.05). D, Heat map representing the overall significant changes in metabolite levels after Br-2′,3′-cAMP treatment. Data are presented as log2 fold change (two-way ANOVA, *P*-value FDR corrected ≤ 0.05).

To characterize plant response to the accumulation of 2′,3′-cAMP, we performed feeding experiments by treating Arabidopsis seedlings growing in liquid cultures with 1 µM of Br-2′,3′-cAMP, a membrane-permeable analog of 2′,3′-cAMP. Samples were harvested after 15 and 30 min and after 1, 6, and 24 h of treatment with either mock solution (control samples) or Br-2′,3′-cAMP (treated samples; Figure 1A). We confirmed the uptake of Br-2′,3′-cAMP using liquid chromatography–mass spectrometry (LC–MS)-based metabolomics (Figure 1B). After 15 min, the samples already showed sufficient levels of Br-2′,3′-cAMP to detect its accumulation in treated seedlings. The level peaked at 30 min, dropped sharply at 1 h, and decreased further at 6 h. Strikingly, no Br-2′,3′-cAMP was detected in the samples taken at 24 h, suggesting a rapid turnover of the compound. In support of the active conversion of 2′,3′-cAMP into adenosine, a decrease in Br-2′,3′-cAMP was accompanied by an increase in the Br-adenosine levels. Again, only traces of Br-adenosine were detected in the samples taken at 24 h, suggesting further decay.

In addition to Br-2′,3′-cAMP and Br-adenosine, metabolomics analysis (Supplemental Tables S1 and S2) identified 142 primary and specialized metabolites, 80 of which were significantly affected by Br-2′,3′-cAMP treatment (two-way analysis of variance [ANOVA], P-value FDR (false discovery date) corrected ≤ 0.05; Figure 1, C and D), and 68 of which were upregulated (Figure 1D). The accumulation of amino acids and proteogenic dipeptides suggests that Br-2′,3′-cAMP treatment induces protein degradation (Figure 1C). Autophagy-dependent accumulation of dipeptides has been reported in plants subjected to heat and dark conditions (Kosmacz et al., 2020; Thirumalaikumar et al., 2020). Autophagy is a known source of amino acids (Hirota et al., 2018). Among the upregulated metabolites, endogenous 2′,3′-cAMP, adenosine-2′-monophosphate, and a 2′,3′-cAMP degradation product were detected in addition to uric acid, which is a product of AMP catabolism (Hauck et al., 2014). This finding suggests that Br-2′,3′-cAMP treatment induces RNA decay, which is a characteristic of stressful conditions (Kosmacz et al., 2018). In summary, 2′,3′-cAMP treatment leads to major metabolic alterations, reminiscent of stress conditions associated with high protein and mRNA turnover rates.

### Transcriptome analysis revealed that Br-2′,3′-cAMP treatment mimics a stress response

**Figure 2:**
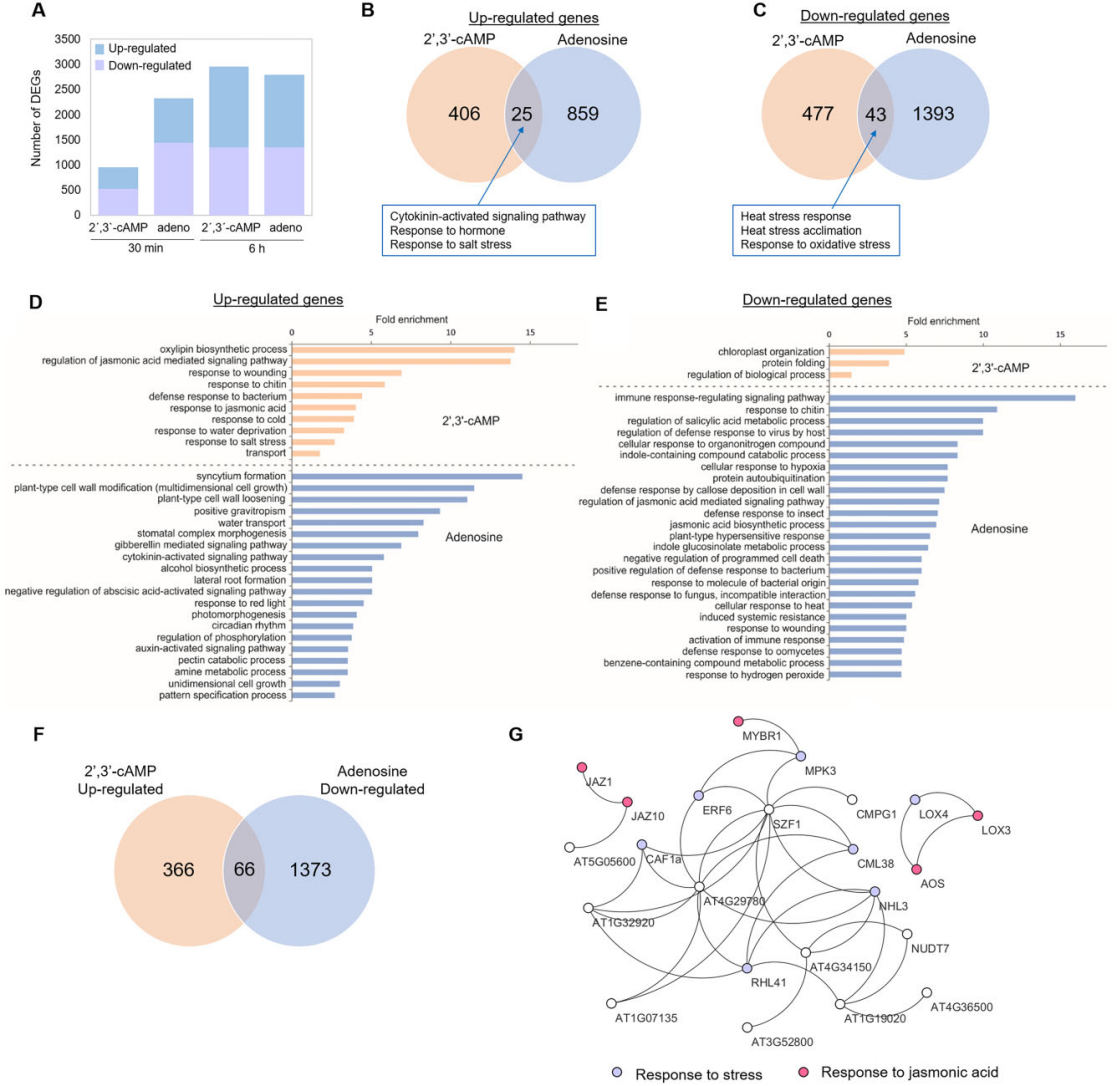
Differential gene expression analysis revealed major transcriptional reprogramming associated with 2′,3′-cAMP and adenosine. A, The number of genes found to be upregulated or downregulated after 30 min and 6 h of treatment. Adeno—corresponds to adenosine. B, Venn diagram of all significantly upregulated genes after 30 min of 2′,3′-cAMP and adenosine treatments. C, Venn diagram of all significantly downregulated genes after 30 min of 2′,3′-cAMP and adenosine treatments. The numbers (B and C) correspond to the DEGs identified as significantly changed compared with the control samples in each experiment. D, Overrepresentation of biological processes in the dataset of upregulated genes in the 2′,3′-cAMP (light orange bars) and adenosine (light blue bars) experiments (30 min time-point). E, Overrepresentation of biological processes in the dataset of downregulated genes in the 2′,3′-cAMP (light orange bars) and adenosine (light blue bars) experiments (30 min time point). In (D and E), overrepresentation is shown as a significant fold enrichment based on the PANTHER overrepresentation test (Mi et al., 2017) using Fisher’s exact test with FDR multiple correction (*P* ≤ 0.05) and *A. thaliana* as the reference organism. F, Venn diagram showing an overlap between downregulated genes after adenosine treatment and upregulated genes after 2′,3′-cAMP treatment. G, A network of enriched genes was retrieved by the STRING database (Szklarczyk et al., 2017) but visualized in Cytoskape. Experimental and database evidence and a low confidence cutoff were used to visualize protein–protein interactions. Gene encoding for proteins involved in stress (violet) and response to jasmonic acid (JA) (pink) are highlighted.

Based on the considerable differences observed at the metabolome level, we expected Br-2′,3′-cAMP treatment to significantly affect the transcriptome. Given that metabolomics analysis revealed adenosine accumulation in response to Br-2′,3′-cAMP treatment, we investigated to what extent the response to Br-2′,3′-cAMP is related to the Br-adenosine build-up. Hence, we performed transcriptional profiling at two time points, 30 min and 6 h, for the Br-2′,3′-cAMP and Br-adenosine treatments. In our analysis, differentially expressed genes (DEGs) were the genes that were significantly upregulated or downregulated (*P*-value adj.  ≤ 0.05) compared with the mock-treated samples. We compared the two datasets, referred to as 2′,3′-cAMP (Br-2′,3′-cAMP versus mock-treated) and adenosine (Br-adenosine versus mock-treated) treatments. We observed high reproducibility between samples (Supplemental Figure S1), with both compounds significantly affecting gene expression (Figure 2A;  Supplemental Tables S3 and S4). Adenosine treatment resulted in 2,322 and 2,798 DEGs at 30 min and 6 h, respectively. Among the 2,322 DEGs measured at 30 min, 547 DEGs were also differentially expressed at 6 h. In contrast, 2′,3′-cAMP treatment resulted in 953 and 2,959 DEGs at 30 min and 6 h, respectively. Among the 953 DEGs measured at 30 min, 439 DEGs were also differentially expressed at 6 h.

The comparison between DEGs identified after adenosine and 2′,3′-cAMP treatments at 30 min (Figure 2, B and C) and 6 h (Supplemental Figure S2) revealed only minor overlap, demonstrating that most of the changes measured in response to 2′,3′-cAMP treatment are specific to 2′,3′-cAMP (Figure 2, B and C). Enrichment analysis of the different biological processes (Fisher’s exact test with an FDR correction, *P*-value ˂ 0.05) was performed using the PANTHER overrepresentation test (PANTHER13.1) with the Gene Ontology (GO) database (Mi et al., 2017). DEGs upregulated by 2′,3′-cAMP and adenosine at 30 min belonged to the groups “cytokinin-activated signaling pathway,” “hormonal response,” and “response to salt stress” (Supplemental Table S5). DEGs downregulated by both 2′,3′-cAMP and adenosine were enriched in genes involved in “heat stress response,” “acclimation,” and “response to oxidative stress” (Supplemental Figure S2 and Supplemental Table S5). 2′,3′-cAMP-specific DEGs induced at 30 min were enriched in transcripts associated with “oxylipin biosynthetic process,” “jasmonic acid (JA) signaling pathways,” and “response to wounding” (Figure 2D;  Supplemental Figure S2 and Supplemental Table S6), whereas 2′,3′-cAMP-downregulated genes comprised multiple transcripts involved in “chloroplast organization” and “protein folding,” including multiple heat shock proteins and genes involved in the heat stress response (Supplemental Figure S2). Approximately 30 min of adenosine treatment resulted in the upregulation of transcripts enriched in biological processes, such as “syncytium formation,” “cell wall modification,” and “hormonal signaling (gibberellin-mediated signaling pathway)” (Figure 2D;  Supplemental Table S6). Moreover, MapMan (Usadel et al., 2009) analysis (Supplemental Figure S3) revealed the overrepresentation of DNA binding with one finger transcription factors, which are involved in biotic stress response, synthesis of seed storage proteins, seed development, photosynthetic processes, and flowering (Wen et al., 2016). In contrast, the downregulated transcripts were highly enriched for “immune response,” “defense regulating pathways,” “indole-containing compound catabolic processes,” and “response to hypoxia” (Figure 2E;  Supplemental Table S7). Interestingly, comparison of the 2′,3′-cAMP-upregulated genes and adenosine-downregulated genes revealed an overlap of 65 genes, mostly involved in stress response and specifically in “response to wounding and JA” (Figure 2, F and G; Supplemental Table S8). This finding suggests that 2′,3′-cAMP and adenosine might have an antagonistic function in the cell in response to stress (e.g. wounding) but a synergistic function in the cytokinin-activated signaling pathway (upregulation) and in response to heat (downregulation). In comparison to the samples induced at 6 h, the main GO categories enriched for the 2′,3′-cAMP-induced transcripts included “photosynthesis,” “hormonal response,” and “stress response,” whereas those enriched for adenosine-induced transcripts included “response to oxidative stress” and “metabolism” (Supplemental Figure S2 and Supplemental Table S9). 2′,3′-cAMP-downregulated transcripts were enriched for “the RNA machinery and processing,” which again supported the role of 2′,3′-cAMP in the regulation of stress-related response (Supplemental Figure S2 and Supplemental Table S10). Finally, adenosine-downregulated transcripts were enriched for “hormonal response to gibberellin” and “hormonal transport”.

Because 2′,3′-cAMP plays a role in the induction of SG formation (Kosmacz et al., 2018), we determined how many DEGs affected by 2′,3′-cAMP treatment contain the prion-like domain (PrLD), which is associated with the formation of liquid–liquid phase separation foci. We focused on the 30 min time-point, as we have previously demonstrated that Br-2′,3′-cAMP treatment for 30 min is sufficient to induce SGs in Arabidopsis seedlings (Kosmacz et al., 2018). Out of the 953 DEGs (at 30 min), 29 encode proteins with a predicted PrLD (Supplemental Figure S4, Supplemental Table S11), including the core component of SGs—Rbp47b. Moreover, proteins such as Auxin-responsive factor 11, PUM5, and PUM6 (pumilio genes), BEL1-like homeodomain 3, and TCP domain protein 10 are on the list.

In summary, we demonstrated that 2′,3′-cAMP and adenosine treatments induced a distinct set of genes, many previously associated with plant stress responses.

### Br-2′,3′-cAMP treatment affects proteins associated with stress response and metabolism

**Figure 3:**
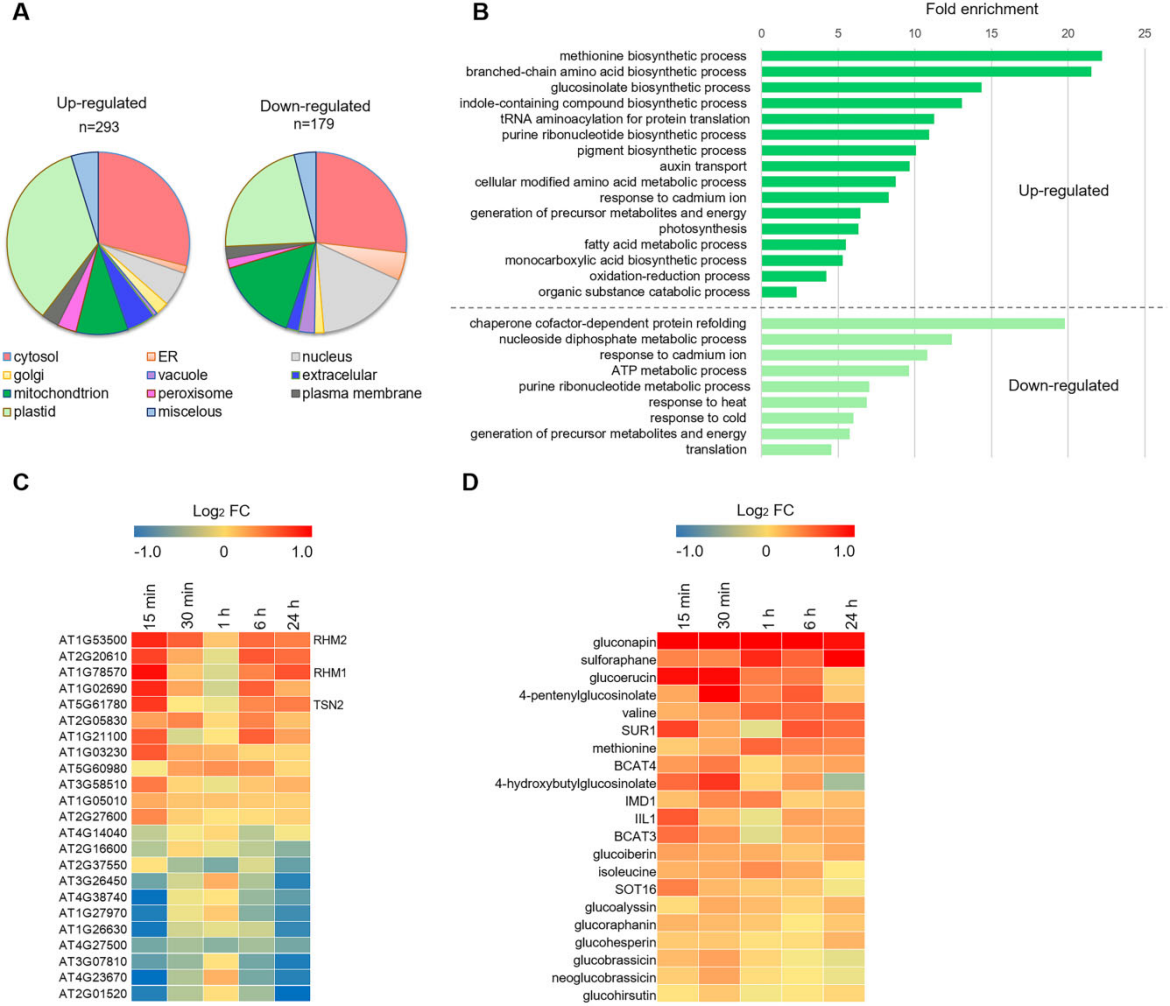
2′,3′-cAMP treatment induces stress-related changes in the proteome of *A. thaliana*. A, The cellular compartment distribution of the identified, significantly upregulated, and downregulated proteins after Br-2′,3′-cAMP treatment. Subcellular localizations for each protein were identified using the SUBA3 database (http://suba3.plantenergy.uwa.edu.au/). B, Enriched biological processes in the dataset of significantly upregulated (dark green) and downregulated (light green) proteins after Br-2′,3′-cAMP treatment. Overrepresentation is shown as a significant fold enrichment based on the PANTHER overrepresentation test (Mi et al., 2017) using Fisher’s exact test with FDR multiple correction (*P* ≤ 0.05) and *A. thaliana* as reference organism. C, Changes in the abundance of SG proteins are presented as heat maps. Significant changes were determined using a two-way ANOVA (*P*-value FDR corrected ≤ 0.05, *n* = 4 biological replicates). D, Heat map represents significantly accumulated putative aliphatic and indole glucosinolate compounds together with glucosinolate biosynthetic enzymes in response to Br-2′,3′-cAMP treatment. Significant changes were determined using a two-way ANOVA (P-value FDR corrected ≤ 0.05, *n* = 4 biological replicates).

To gain additional insights into the response to Br-2′,3′-cAMP treatment, we analyzed the changes in the abundance of proteins at all five time-points. Statistical analysis identified 472 differentially abundant proteins (two-way ANOVA, *P*-value FDR corrected ≤ 0.05; Supplemental Table S1). Subcellular localization analysis revealed an enrichment of plastid and cytosol localization among upregulated proteins whereas an enrichment of nuclear and cytosol localization among downregulated proteins (Figure 3A). Functional analysis of upregulated proteins (PANTHER) identified functional categories, such as “amino acids, glucosinolates, nucleotide, and pigment biosynthesis,” “photosynthesis,” and “auxin transport.” Downregulated proteins, analogous to downregulated transcripts, were enriched for proteins associated with protein folding, energy metabolism, response to heat, and translation (Figure 3B).

Because 2′,3′-cAMP accumulates in response to wounding (Van Damme et al., 2014) and Br-2′,3′-cAMP treatment induces genes associated with jasmonate synthesis and signaling (see above), we were intrigued by the accumulation of glucosinolate biosynthetic enzymes. Glucosinolates are sulfur-containing specialized metabolites that contribute to plant defense against pests, and glucosinolate accumulation depends on jasmonate signaling (Mitreiter and Gigolashvili, 2021). To further investigate our observation, we searched for glucosinolates within the unknown features of our metabolomics dataset. Annotation based on a previous study (Wu et al., 2018) led to the identification of 13 putative aliphatic and indole glucosinolate compounds, 11 of which accumulated in response to the Br-2′,3′-cAMP treatment (two-way ANOVA, *P* ≤ 0.05; Supplemental Table S12; Figure 3D). Whereas glucosinolate accumulation peaks at 15–30 min of Br-2′,3′-cAMP treatment, sulforaphane, a product of glucosinolate break-down, gradually builds up and reaches its maximum accumulation after 24 h. Moreover, the list of 2′,3′-cAMP-responsive metabolites includes glucosinolate precursor amino acids (methionine, valine, and isoleucine), which supports the accumulation of enzymes involved in branch-chain amino acid and methionine metabolism. However, note that glucosinolate accumulation preceded the accumulation of amino acids. Finally, we investigated whether glucosinolate biosynthetic enzymes induced by Br-2′,3′-cAMP at the protein level are also induced at the transcript level and found that this was not the case.

We also found that a crucial enzyme for aliphatic glucosinolate synthesis, SUR1, which was previously found among proteins sequestered into cytosolic SG (Kosmacz et al., 2019; Kosmacz and Skirycz, 2020), is induced by Br-2′,3′-cAMP treatment. In addition to SUR1, 22 proteins previously reported to localize to SG (Figure 3C;  Supplemental Table S1) respond to Br-2′,3′-cAMP treatment: 12 upregulated and 11 downregulated. The list of Br-2′,3′-cAMP-responsive proteins includes RHM2, RHM1 (Kosmacz et al., 2019), and tudor staphylococcal nuclease 2 (TSN2) (Gutierrez-Beltran et al., 2015), providing additional evidence that links 2′,3′-cAMP and stress response at the SG level. Interestingly, we also observed accumulation of glucosinolate biosynthetic enzymes that correlated with accumulation of glucosinolate compounds (Figure 3D), which supports that observed changes are related to stress response.

Finally, joined clustering analysis of protein and metabolite data delineated several patterns of accumulation (Supplemental Figure S5 and Supplemental Table S13). Notably, most proteins and metabolites, 257 out of 563, were characterized by the largest change measured at the early 15-min and subsequently late 6- and 24-h time-points, with more minor or no changes at 30 min and 1 h (Supplemental Figure S5; Supplemental Table S13). In comparison, Br-2′,3′-cAMP peaked at 30 min, followed by a gradual decline, with no compound detected at 24 h. We attribute this perplexing behavior to endogenous 2′,3′-cAMP. 2′,3′-cAMP level was induced by the Br-2′,3′-cAMP treatment, with the highest accumulation measured at 6 and 24 h. Among the proteins that follow the pattern of Br-2′,3′-cAMP/2′,3′-cAMP accumulation are those located in cluster 4 of 26 downregulated proteins and metabolites (Supplemental Figure S5A), such as chaperones, proteasome subunit (PAG1), or GRF10 protein (14-3-3-like protein GF14 epsilon) from the brassinosteroid pathway.

### 2′,3′-cAMP treatment induces movement of PBs

**Figure 4:**
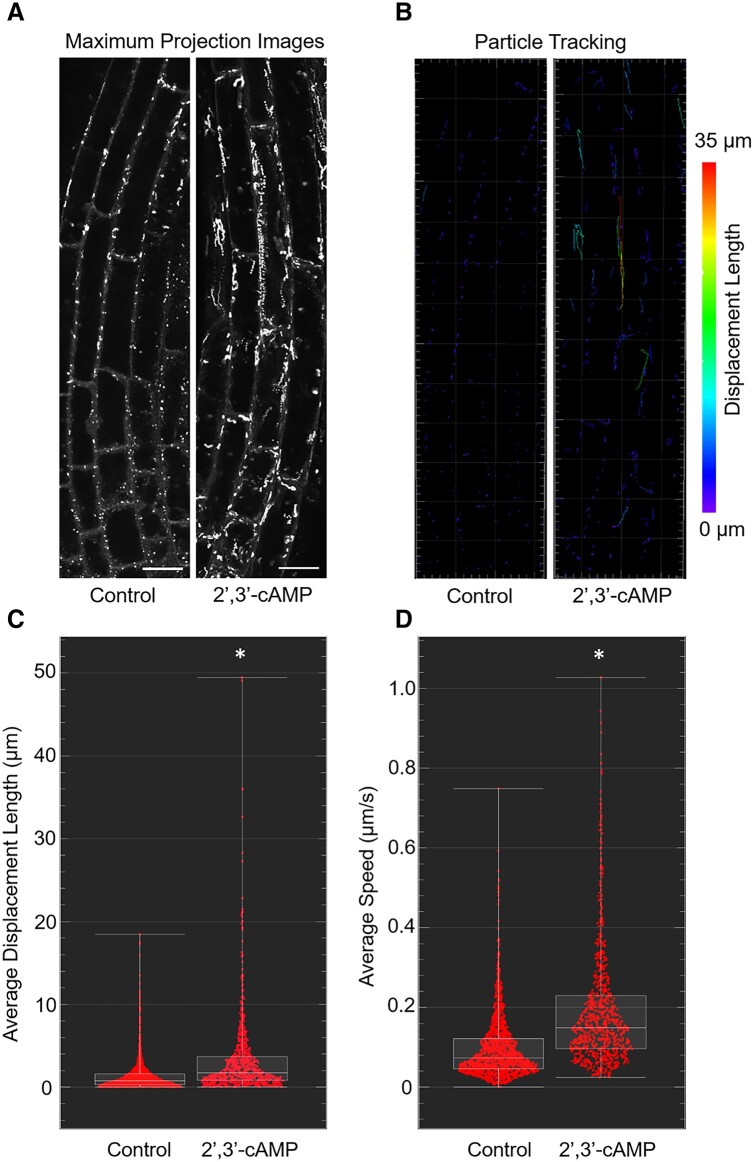
Br-2′,3′-cAMP treatment induces motility of PBs. A, Maximum projection images from 61 time-points collected from GFP-DCP1 Arabidopsis seedlings under control and after Br-2′,3′-cAMP treatment. Scale bar = 10 µm. B, Particle tracking in the control and Br-2′,3′-cAMP-treated cells. Scale represents the color code for the displacement length for each PB. C, The average displacement length of the PBs in the cell is expressed in micrometer. D, The average speed of PB movement in the control and Br-2′,3′-cAMP-treated seedlings. Speed is expressed in µm/s. For C and D, control *n* = 1,583 and 2′,3′-cAMP *n* = 898. Asterisk in C and D indicates significant differences defined by student′s *t* test, *P* ≤ 0.05.

Given that SGs are highly connected with PBs and that 2′,3′-cAMP is closely related to RNA metabolism (being its degradation product), we investigated whether 2′,3′-cAMP can also affect PBs. The main function of PBs is translational repression and mRNA decay (Kedersha et al., 2005; Xu and Chua, 2011). However, both PBs and SGs continuously interact (Anderson and Kedersha, 2009) and are often transiently linked to each other (Eisinger-Mathason et al., 2008; Buchan et al., 2012). In contrast with SGs, PBs are always present in the cell, regardless of stress conditions; however, stress may affect the dynamic of PBs. To evaluate this feature, we focused on GFP-decapping protein 1 (DCP1) protein, which is a well-known PB marker previously used for co-localization studies with SGs (Gutierrez-Beltran et al., 2015), and determined whether the treatment affects PB dynamics. Using confocal microscopy and aligning t-stags (Figure 4, A and B), we followed the movement of PBs over time. Particle tracking showed that Br-2′,3′-cAMP treatment significantly induced displacement length (Figure 4C;  Supplemental Table S14) and PB movement speed (Figure 4D) compared with the control samples (Supplemental Table S15). Our experiments indicate that 2′,3′-cAMP treatment not only induces genes that contain PrLD but also affects proteins known to localize to SG/PBs. This could explain why PB dynamics are affected, further suggesting a role of 2′,3′-cAMP in the regulation of PB motility.

## Discussion

Given that 2′,3′-cAMP has been detected in plants (Pabst et al., 2010), responds to stress conditions (Van Damme et al., 2014), and is a facilitator of SG formation (Kosmacz et al., 2018), further characterizing its function at different molecular levels is necessary. Our multi-omics approach (Moreno et al., 2021) is a valuable strategy to gain further insights at different molecular levels. This study compared cellular responses in plants at the metabolome, proteome, and transcriptome levels upon membrane-permeable 2′,3′-cAMP (Br-2′,3′-cAMP) treatment.

In animal cells, 2′,3′-cAMP is metabolized to adenosine. Our metabolomics analysis revealed that after 30 min of treatment in plants, the level of Br-2′,3′-cAMP decreases and that of Br-adenosine increases (Figure 1A), suggesting the existence of the 2′,3′-cAMP adenosine salvage pathway in plants. Notably, these results are in line with previous findings showing that putative 2′,3′-cNMP cyclic phosphodiesterase, an enzyme involved in the digestion of cNMPs, exists in *Arabidopsis* *thaliana* (Genschik et al., 1997). This study further supports the finding that 2′,3′-cAMP is metabolized in plant cells and is a part of the adenosine salvage pathway that has only been described to exist in mammalian cells (Jackson et al., 2009). Interestingly, the nucleoside transporter (ENT) is important for regulating the levels of 2′,3′-cAMP and adenosine in plant cells. Transgenic Arabidopsis plants with either low or high expression of the ENT are characterized by concomitant changes in the 2′,3′-cAMP and adenosine levels (Bernard et al., 2011). This finding again suggests that the concentration of those two compounds is tightly regulated by the presence of adenosine pathway components.

To distinguish between 2′,3′-cAMP- and adenosine-specific changes, we first evaluated the transcriptional response to 2′,3′-cAMP and adenosine (Br-adenosine). Transcriptomics data revealed that although the genes upregulated by 2′,3′-cAMP and those downregulated by adenosine overlapped, the overall changes caused by these two compounds were significantly different. This result points toward the distinct functions of 2′,3′-cAMP and adenosine. Genes and proteins affected by 2′,3′-cAMP are involved in several biological processes but are especially enriched in known stress markers, such as ATG10 (AT3G07525), which is involved in the formation of autophagy vesicles; JAZ1 (AT1G19180) and JAZ10 (AT5G13220), which are central components of jasmonate signaling; critical stress kinase—MPK3 (AT3G45640), transcription factors such as MYB44 (AT5G67300); and markers of heat response such as HSP70 (AT3G12580) and MBFC1 (AT3G24500). Furthermore, metabolomics analysis revealed the accumulation of known stress markers, such as glucosinolates, RNA-degradation products, and proteogenic dipeptides (Figure 1C) (Doppler et al., 2019; Thirumalaikumar et al., 2020; Moreno et al., 2021). The latter is especially interesting given the regulatory roles of dipeptides, such as in the regulation of enzymes of the central carbon metabolism. Dipeptide feeding affects metabolic fluxes, leading to changes in the metabolite pools impacting plant growth under oxidative stress (Moreno et al., 2021) and diauxic shift transition in yeast (Luzarowski et al., 2021). Hence, we speculate that the 2′,3′-cAMP-related accumulation of dipeptides contributes to the 2′,3′-cAMP response.

2′,3′-cAMP binds to the RNA-binding motifs (RRMs) of Rbp47b, an SG assembly protein (Maruri-Lopez et al., 2021). In line with the binding data, 2′,3′-cAMP treatment facilitates SG formation (Kosmacz et al., 2018). RRM domains are not restricted to the Rbp47b protein. In fact, RRM is one of the most abundant protein domains in eukaryotes; it is also present in proteins associated with different facets of RNA metabolism, including RNA sequestration to non-membrane aggregates, such as SG. Among the 31 recently predicted 2′,3′-cAMP targets, 7 contain the RRM domain (Zuhlke et al., 2021), including a plastidial protein CP29, which is a component of plastidial SGs (Chodasiewicz et al., 2020). Here, we demonstrated that 2′,3′-cAMP, in addition to binding to the core SG proteins, affects the abundance of proteins that sequester into SGs in response to heat condition (Kosmacz et al., 2019), such as TSN (Gutierrez-Beltran et al., 2015). Notably, TSN is also found in PBs; in addition to its scaffolding role, TSN is involved in mRNA decapping (Gutierrez-Beltran et al., 2015). Intriguingly, we showed that 2′,3′-cAMP increases the motility of PBs. PB movement depends on myosin (Steffens et al., 2014) and on interactions with SGs. Various components are known to be exchanged between these two foci (Kedersha et al., 2005); therefore, any effect on SGs may directly affect PBs.

**Figure 5:**
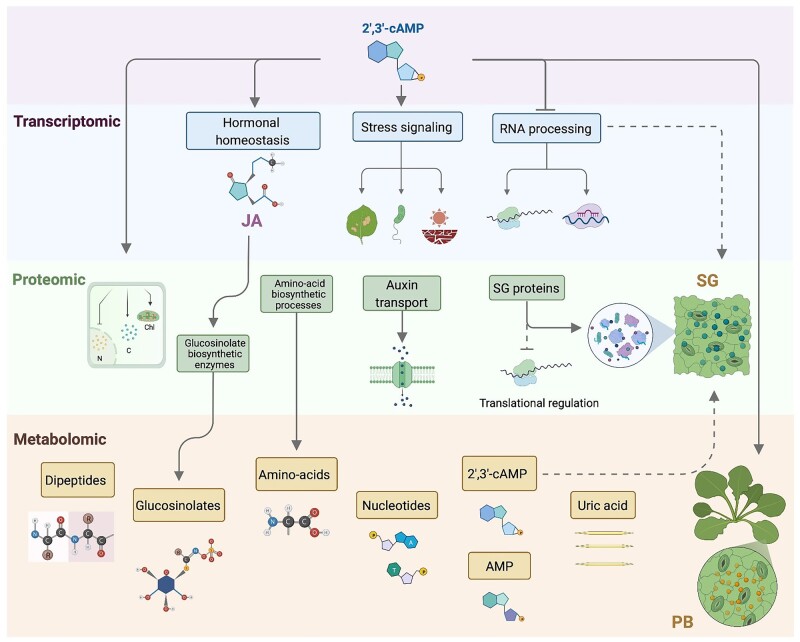
2′,3′-cAMP treatment resembles stress responses. Schematic representation of the effects of 2′,3′-cAMP (purple panel) treatment in *A. thaliana* at the transcriptomics, proteomics, and metabolomics levels. Transcriptomics data (blue panel). Differential gene expression analysis showed the 2′,3′-cAMP upregulation of genes involved in JA homeostasis and stress responses (e.g. wounding, defense to bacteria, and water deprivation), whereas those genes involved in RNA machinery and processing were downregulated during 2′,3′-cAMP treatment. Proteomics data (green panel) revealed that 2′,3′-cAMP triggered stress-related changes in the *A. thaliana* proteome. Briefly, the accumulation of proteins involved in amino acid biosynthetic pathways and auxin transport increased upon 2′,3′-cAMP treatment, in contrast to translational machinery, which was downregulated. 2′,3′-cAMP treatment also led to the accumulation of key SG proteins and induced PB movement. Metabolites (orange panel) such as dipeptides, amino acids, nucleotides, and RNA-degradation products showed accumulation after 2′,3′-cAMP treatment. Arrows indicate upregulation, and bars depict downregulation. Dotted lines denote the associated process.

In summary, 2′,3′-cAMP treatment affects the levels of hundreds of transcripts, proteins, and metabolites, many of which have been previously associated with plant stress response (Figure 5). The response is rapid and specific, occurring within the first 15–30 min of 2′,3′-cAMP treatment. Future works will focus on further characterization of the downstream responses, including changes in PB motility and dipeptide accumulation.

## Materials and methods

### Plant growth conditions and feeding experiments

Arabidopsis (*A.* *thaliana*) Col-0 seedlings were grown in liquid Murashige and Skoog (MS) medium (Murashige and Skoog, 1962) supplied with 1% *v/v* sucrose in continuous light. The medium was changed after 7 d, and treatment with 1-µM Br-2′,3′-cAMP (Biolog Life Science Institute, Bremen, Germany) was performed after 3 d (on Day 10). Approximately 1 µM was used based on low-to-mid micrometer concentrations of endogenous 2′,3′-cAMP previously measured in Arabidopsis native lysate (Kosmacz et al., 2018). As the control, the seedlings were treated with water (in which the compound was dissolved). The seedlings were then harvested after 15 and 30 min and after 1, 6, and 24 h of treatment, rapidly dried on paper, and frozen in liquid nitrogen. Dry plant material was obtained after dry vacuuming for 3 d. For transcriptome analysis, the same experiment using the same conditions with 1 µM Br-adenosine (Sigma-Aldrich, St Louis, MO, USA) was performed.

### Metabolite and protein extraction

The protocol for the extraction of molecules was adjusted from Salem et al. (2017). Using 10 mg of dried tissue powder, macromolecules were extracted using a methyl tert-butyl ether (MTBE)/methanol/water solvent system, which separates the molecules into pellet (proteins), organic (lipids), and aqueous phases (primary and secondary metabolites). Equal volumes of the collected fractions were dried using a centrifugal evaporator and stored at −80°C before metabolomics and proteomics analyses.

### LC‒MS metabolomics for secondary metabolite identification

The dried aqueous phase was measured using ultra-performance LC coupled to an exactive mass spectrometer (Thermo Fisher Scientific, Bremen, Germany) in positive and negative ionization modes. The method was reproduced from Giavalisco et al. (2011). However, the spectra were recorded in full-scan mode only. Data processing was performed using REFINER MS version 10.5 (GeneData; http://www.genedata.com) and included peak detection, chemical noise subtraction, retention-time (RT) alignment, and integration of isotopic peaks into peak clusters. Metabolite features were annotated using an in-house reference compound library allowing 10 ppm *m/z* and 0.1 min RT deviations. The library comprises authentic chemical standards, which were run using the method described above. The obtained chromatograms were used to extract information on the main adduct and RT. The library also comprises phenylpropanoids, flavonols, and glucosinolates, which were annotated based on fragmentation and elemental formula information, as described by (Wu et al., 2018), using identical instruments and methods.

### LC–MS/MS for protein and data analysis

Protein pellets formed in the MTBE-based extraction method were solubilized in 100 μL of urea–thiourea buffer (6-M urea and 2-M thiourea in 40-mM ammonium bicarbonate). Protein content was determined using a Bradford assay (Carl Roth, Karlsruhe, Germany). Approximately 40 µg of protein was treated with 5 mM of dithiothreitol for 30 min at room temperature, followed by cysteine alkylation with 15-mM iodoacetamide for 20 min at room temperature in the dark. Enzymatic digestion of proteins using LysC/trypsin mix (Promega, Fitchburg, WI, USA) was then performed according to the technical manual. After digestion, samples were acidified with trifluoroacetic acid (TFA) to pH < 2. Peptides were desalted using C18 Empore extraction discs (3 M, Maplewood, MN, USA) and STAGE tips (Rappsilber et al., 2003) and dried to ∼4 µL using a centrifugal evaporator. Samples were stored at −80°C until measurement. Dried peptides were solubilized in loading buffer (2% ACN, 0.2% TFA), and an equivalent of 0.8–1.0 µg of peptides was separated using a reversed-phase column and analyzed on a Q-Exactive Plus or Q-Exactive HF spectrometer (Thermo Fisher Scientific, Waltham, MA, USA). MaxQuant version 1.6.0.16 (Cox and Mann, 2008) and its built-in search engine Andromeda (Cox et al., 2011) were used to analyze the raw proteomics data. For protein annotation, the *A. thaliana* TAIR10 annotations (Arabidopsis TAIR database version 10, The Arabidopsis Information Resource, www.Arabidopsis.org [updated in December 2017]) combined with the search engine Andromeda were used. The search also included a contaminant database that can be found in any MaxQuant installation folder or directly downloaded from the developer’s official website. Contaminants and decoy hits were removed from each dataset. The settings for MaxQuant analysis were as follows: trypsin and lysine were selected as digesting enzymes, two missed cleavages were allowed, fixed modification was set to carbamidomethylation (cysteine), and oxidation of methionine was set as a variable modification. Spectra were also searched against a decoy database of the *A. thaliana* proteome, and results were filtered to obtain an FDR below 1% on the protein level. The “label-free quantification” (LFQ) and “match between runs” options were selected. A minimum peptide length of six amino acids was used. Quantification was performed for proteins with a minimum of one unique and one razor peptide. Known contaminants, such as keratins, were removed from further analysis. Furthermore, at least two unique peptides were required per protein group. LFQ intensities were used in all analyses performed in this study.

### RNA extraction and RNAseq analysis

An RNA extraction kit (Macherey-Negel) was used to extract total RNA from 10 mg of lyophilized tissue, followed by quality assessment using Bioanalyzer RNA 6000 Nano (Agilent, Santa Clara, CA, USA). RNAseq analysis was performed by Lexogen GmbH (Lexogen, Vienna, Austria) using QuantSeq 3′-mRNA library preparation and QuantSeq 3′-UTR NextSeq SR75 sequencing. An integrated data analysis was performed by Lexogen (STAR aligner), where the reads were mapped against the *A. thaliana* reference genome (Araport11), read counts were determined, and differential expression was computed using DESeq2 in R (Love et al., 2014). To perform RNAseq analysis, two biological replicates (one biological sample corresponds to independently treated flask of Arabidopsis seedlings) for two time-points of 30 min and 6 h were used in each experiment. MapMan software (Usadel et al., 2009) was used to visualize perturbations in gene expression.

### Statistical analysis

GeneData-derived raw metabolite intensities were normalized to the median intensity of all mass features detected in a given chromatogram. MaxQuant-derived LFQ intensities were used for further analysis. Both metabolite and protein data were subjected to log_2_ transformation prior to two-way ANOVA analysis implemented in the MeV software (Howe et al., 2011) using treatment (treated versus untreated) and time (15 min, 30 min, 1 h, 6  h, and 24 h) as variables. The obtained *P*-values were subjected to FDR correction to select metabolites and proteins significantly affected by the treatment. The MeV software (Howe et al., 2011) was used to obtain heat maps. Differential gene expression was computed using DESeq2 in R (Love et al., 2014). The obtained *P*-values were FDR-corrected. For the proteomics and transcriptomics analyses, a fold enrichment analysis was performed using the PANTHER overrepresentation test (PANTHER13.1) based on Fisher′s exact test with FDR correction *P* ≤ 0.05. Subcellular localization analyses were retrieved from the SUBA3 database (Tanz et al., 2013). Clustering of proteomics and metabolomics data was also performed using the MeV software. For Figure 4, student’s *t* test was used, *P* ≤ 0.05 was used to define significant changes.

### PrLD analysis

PrLD analysis was performed using the PLAAC software, where the amino acid sequences of all significant DEGs from 30 min of Br-2′,3′-cAMP treatment were uploaded. Data are present in Supplemental Table S10.

### Data deposition

The MS proteomics data have been deposited to the ProteomeXchange Consortium via the PRIDE (Perez-Riverol et al., 2016) partner repository with the dataset identifier PXD028365. RNAseq raw data have been deposited to the National Center for Biotechnology Information (NCBI) repository with the number PRJNA769461.

### PB dynamic assessment under confocal microscope

Arabidopsis seeds expressing GFP-tagged DCP1 (Gutierrez-Beltran et al., 2015), the PB marker, were kindly provided by Dr Emilio Gutierrez-Beltran. Plants were grown for 5–7 d on MS media supplied with 1% sucrose. On the day of the experiment, seedlings were moved to an Eppendorf tube and incubated either with water (control) or with 100 µM Br-2′,3′-cAMP for 30 min. After incubation, the seedlings were observed under a confocal microscope (Leica TCS SP8) using the x, y, t function. GFP was excited using a 488-nm laser (10% laser intensity), hybrid detection and emission was obtained between 500 and 600 nm. Images were collected every 2 s for a total of 69 images, which were used for video assembly. The PB displacement and displacement speed were calculated using the IMARIS software (https://imaris.oxinst.com/).

### Accession numbers

Sequence data from this article can be found in the GenBank/EMBL data libraries under accession numbers presented in Supplemental Tables S1 and S3.

## Supplemental data

The following materials are available in the online version of this article.

**Supplemental Figure S1.** Clustering of transcriptomics profiles between replicates of treated and untreated seedlings.

**Supplemental Figure S2.** Overrepresentation of the biological process in a set of upregulated and downregulated specific genes in 2′,3′-cAMP (orange bars) and adenosine experiments (blue bars).

**Supplemental Figure S3.** MapMan representation of transcriptional perturbations in 30 min time-points for Br-2′,3′-cAMP (A) and Br-adenosine treatments (B).

**Supplemental Figure S4.** Transcriptome-wide analysis identified 29 DEGs with PrLDs.

**Supplemental Figure S5.** Co-expression clustering between proteins and metabolites that are downregulated or upregulated by Br-2′,3′-cAMP treatment.

**Supplemental Table S1.** Protein and metabolite analysis of the 2′,3′-cAMP experiment.

**Supplemental Table S2.** Information on the metabolites annotation.

**Supplemental Table S3.** All genes annotated in RNAseq experiment after 30 min and 6 h of treatment with Br-2′,3′-cAMP and Br-adenosine.

**Supplemental Table S4**. Number of all DEGs in two experiments after 30 min and 6 h of treatment.

**Supplemental Table S5.** Functional enrichment test for overlapping genes between up-regulated/downregulated DEGs at 30 min of 2′,3′-cAMP and Adenosine treatment (based on Fisher’s test).

**Supplemental Table S6**. Panther overrepresentation test for upregulated genes specific for 30 min 2′,3′-cAMP and Adenosine treatment based on Fisher’s test.

**Supplemental Table S7**. Panther overrepresentation test for downregulated genes specific for 30 min 2′,3′-cAMP and Adenosine treatment based on Fisher’s test.

**Supplemental Table S8.** Functional enrichment test for overlapping genes between upregulated DEGs in 30 min of 2′,3′-cAMP and downregulated DEGs Adenosine treatment (based on Fisher’s test).

**Supplemental Table S9**. Panther overrepresentation test for upregulated genes specific for 6 h 2′,3′-cAMP and Adenosine treatment based on Fisher’s test.

**Supplemental Table S10**. Panther overrepresentation test for downregulated genes specific for 6 h of 2′,3′-cAMP and Adenosine treatment based on Fisher’s test.

**Supplemental Table S11**. Prion Domain analysis of 953 2′,3′-cAMP affected DEGs.

**Supplemental Table S12.** Glucosinolates measured in the 2′,3′-cAMP experiment.

**Supplemental Table S13.** Co-expression clustering of proteins and metabolites which are up- or downregulated by 2′,3′-cAMP.

**Supplemental Table S14.** Track Displacement Length of PBs under control and 2′,3′-cAMP treatment.

**Supplemental Table S15.** Track speed mean under control and 2′,3′-cAMP treatment.

## Funding

Work was funded by Max Planck Society, KAUST University and Boyce Thompson Institute.

*Conflict of interest statement*: None declared.

## Supplementary Material

kiac013_Supplementary_Data

## Notes

M.C., O.K., and J.C.M. performed the feeding experiments for Br-2′,3′-cAMP and Br-adenosine; M.C. and O.K. prepared the samples for metabolomics, transcriptomics, and proteomics analyses; M.G. ran and annotated the proteomics samples and deposited the data to PRIDE. Aleksandra S. performed the annotation of metabolomics data and analysis; M.C., A.D.L.N., and Aleksandra S. analyzed the transcriptomics and proteomics data; I.M.-L. performed data mining and prepared the summary figure; M.C. performed confocal microscopy for PBs; R.I.M. provided help and organized the supplementary material; Arun S. performed measurements on PB dynamics; Aleksandra S. and M.C. conceived the project; A.D.L.N. submitted RNAseq raw data to the NCBI repository; and M.C. wrote the paper with input from Aleksandra S., J.C.M., and A.D.L.N. All authors contributed to the final version of this manuscript.

The author responsible for distribution of materials integral to the findings presented in this article in accordance with the policy described in the Instructions for Authors (https://academic.oup.com/plphys/pages/general-instructions) is Monika Chodasiewicz (monika.chodasiewicz@kaust.edu.sa).

## References

[kiac013-B1] Alqurashi M , Gehring C, Marondedze C (2016) Changes in the Arabidopsis thaliana proteome implicate cAMP in biotic and abiotic stress responses and changes in energy metabolism. Int J Mol Sci 17: 852 10.3390/ijms17060852 PMC4926386 27258261

[kiac013-B2] Anderson P , Kedersha N (2009) RNA granules: post-transcriptional and epigenetic modulators of gene expression. Nat Rev Mol Cell Biol 10: 430–436 19461665 10.1038/nrm2694

[kiac013-B3] Azarashvili T , Krestinina O, Galvita A, Grachev D, Baburina Y, Stricker R, Evtodienko Y, Reiser G (2009) Ca2+-dependent permeability transition regulation in rat brain mitochondria by 2',3'-cyclic nucleotides and 2',3'-cyclic nucleotide 3'-phosphodiesterase. Am J Physiol Cell Physiol 296: C1428–C1439 19357238 10.1152/ajpcell.00006.2009

[kiac013-B4] Bernard C , Traub M, Kunz HH, Hach S, Trentmann O, Mohlmann T (2011) Equilibrative nucleoside transporter 1 (ENT1) is critical for pollen germination and vegetative growth in Arabidopsis. J Exp Bot 62: 4627–4637 21642237 10.1093/jxb/err183 PMC3170557

[kiac013-B5] Buchan JR , Capaldi AP, Parker R (2012) TOR-tured yeast find a new way to stand the heat. Mol Cell 47: 155–157 22841000 10.1016/j.molcel.2012.07.005

[kiac013-B6] Catozzi S , Di-Bella JP, Ventura AC, Sepulchre JA (2016) Signaling cascades transmit information downstream and upstream but unlikely simultaneously. BMC Syst Biol 10: 84 27561377 10.1186/s12918-016-0303-2 PMC5000522

[kiac013-B7] Chantarachot T , Bailey-Serres J (2018) Polysomes, stress granules, and processing bodies: a dynamic triumvirate controlling cytoplasmic mRNA fate and function. Plant Physiol 176: 254–269 29158329 10.1104/pp.17.01468 PMC5761823

[kiac013-B8] Chodasiewicz M , Sokolowska EM, Nelson-Dittrich AC, Masiuk A, Beltran JCM, Nelson ADL, Skirycz A (2020) Identification and characterization of the heat-induced plastidial stress granules reveal new insight into Arabidopsis stress response. Front Plant Sci 11: 595792 33224174 10.3389/fpls.2020.595792 PMC7674640

[kiac013-B9] Cox J , Mann M (2008) MaxQuant enables high peptide identification rates, individualized p.p.b.-range mass accuracies and proteome-wide protein quantification. Nat Biotechnol 26: 1367–1372 19029910 10.1038/nbt.1511

[kiac013-B10] Cox J , Neuhauser N, Michalski A, Scheltema RA, Olsen JV, Mann M (2011) Andromeda: a peptide search engine integrated into the MaxQuant environment. J Proteome Res 10: 1794–1805 21254760 10.1021/pr101065j

[kiac013-B11] Doppler M , Kluger B, Bueschl C, Steiner B, Buerstmayr H, Lemmens M, Krska R, Adam G, Schuhmacher R (2019) Stable isotope-assisted plant metabolomics: investigation of phenylalanine-related metabolic response in wheat upon treatment with the fusarium virulence factor deoxynivalenol. Front Plant Sci 10: 1137 31736983 10.3389/fpls.2019.01137 PMC6831647

[kiac013-B12] Eisinger-Mathason TS , Andrade J, Groehler AL, Clark DE, Muratore-Schroeder TL, Pasic L, Smith JA, Shabanowitz J, Hunt DF, Macara IG, et al (2008) Codependent functions of RSK2 and the apoptosis-promoting factor TIA-1 in stress granule assembly and cell survival. Mol Cell 31: 722–736 18775331 10.1016/j.molcel.2008.06.025 PMC2654589

[kiac013-B13] Genschik P , Hall J, Filipowicz W (1997) Cloning and characterization of the Arabidopsis cyclic phosphodiesterase which hydrolyzes ADP-ribose 1'',2''-cyclic phosphate and nucleoside 2',3'-cyclic phosphates. J Biol Chem 272: 13211–13219 9148938 10.1074/jbc.272.20.13211

[kiac013-B55] **Giavalisco P, Li Y, Matthes A, Eckhardt A, Hubberten H-A, Hesse H, Segu S, Hummel J, Köhl K, Willmitzer L** (2011) Elemental formula annotation of polar and lipophilic metabolites using ^13^C, ^15^N and ^34^S isotope labelling, in combination with high-resolution mass spectrometry. Plant J 10.1111/j.1365-313X.2011.04682.x 21699588

[kiac013-B14] Gutierrez-Beltran E , Moschou PN, Smertenko AP, Bozhkov PV (2015) Tudor staphylococcal nuclease links formation of stress granules and processing bodies with mRNA catabolism in Arabidopsis. Plant Cell 27: 926–943 25736060 10.1105/tpc.114.134494 PMC4558657

[kiac013-B15] Hauck OK , Scharnberg J, Escobar NM, Wanner G, Giavalisco P, Witte CP (2014) Uric acid accumulation in an Arabidopsis urate oxidase mutant impairs seedling establishment by blocking peroxisome maintenance. Plant Cell 26: 3090–3100 25052714 10.1105/tpc.114.124008 PMC4145134

[kiac013-B16] Hirota T , Izumi M, Wada S, Makino A, Ishida H (2018) Vacuolar protein degradation via autophagy provides substrates to amino acid catabolic pathways as an adaptive response to sugar starvation in Arabidopsis thaliana. Plant Cell Physiol 59: 1363–1376 29390157 10.1093/pcp/pcy005

[kiac013-B17] Howe EA , Sinha R, Schlauch D, Quackenbush J (2011) RNA-Seq analysis in MeV. Bioinformatics 27: 3209–3210 21976420 10.1093/bioinformatics/btr490 PMC3208390

[kiac013-B18] Huising MO , van der Aa LM, Metz JR, de Fatima Mazon A, Kemenade BM, Flik G (2007) Corticotropin-releasing factor (CRF) and CRF-binding protein expression in and release from the head kidney of common carp: evolutionary conservation of the adrenal CRF system. J Endocrinol 193: 349–357 17535873 10.1677/JOE-07-0070

[kiac013-B19] Jackson EK (2016) Discovery and roles of 2',3'-cAMP in biological systems. Handb Exp Pharmacol 238: 229–252 10.1007/164_2015_40 26721674

[kiac013-B20] Jackson EK , Ren J, Mi Z (2009) Extracellular 2',3'-cAMP is a source of adenosine. J Biol Chem 284: 33097–33106 19801686 10.1074/jbc.M109.053876 PMC2785151

[kiac013-B21] Jang GJ , Jang JC, Wu SH (2020) Dynamics and functions of stress granules and processing bodies in plants. Plants (Basel) 9: 1122 10.3390/plants9091122 PMC7570210 32872650

[kiac013-B22] Kedersha N , Stoecklin G, Ayodele M, Yacono P, Lykke-Andersen J, Fritzler MJ, Scheuner D, Kaufman RJ, Golan DE, Anderson P (2005) Stress granules and processing bodies are dynamically linked sites of mRNP remodeling. J Cell Biol 169: 871–884 15967811 10.1083/jcb.200502088 PMC2171635

[kiac013-B23] Kosmacz M , Gorka M, Schmidt S, Luzarowski M, Moreno JC, Szlachetko J, Leniak E, Sokolowska EM, Sofroni K, Schnittger A, et al (2019) Protein and metabolite composition of Arabidopsis stress granules. New Phytol 222: 1420–1433 30664249 10.1111/nph.15690

[kiac013-B24] Kosmacz M , Luzarowski M, Kerber O, Leniak E, Gutierrez-Beltran E, Moreno JC, Gorka M, Szlachetko J, Veyel D, Graf A, et al (2018) Interaction of 2',3'-cAMP with Rbp47b plays a role in stress granule formation. Plant Physiol 177: 411–421 29618637 10.1104/pp.18.00285 PMC5933139

[kiac013-B25] Kosmacz M , Skirycz A (2020) The Isolation of Stress Granules From Plant Material. Curr Protoc Plant Biol 5: e20118 32946676 10.1002/cppb.20118

[kiac013-B26] Kosmacz M , Sokolowska EM, Bouzaa S, Skirycz A (2020) Towards a functional understanding of the plant metabolome. Curr Opin Plant Biol 55: 47–51 32224339 10.1016/j.pbi.2020.02.005

[kiac013-B27] Love MI , Huber W, Anders S (2014) Moderated estimation of fold change and dispersion for RNA-seq data with DESeq2. Genome Biol 15: 550 25516281 10.1186/s13059-014-0550-8 PMC4302049

[kiac013-B28] Ludwig N , Yerneni SS, Menshikova EV, Gillespie DG, Jackson EK, Whiteside TL (2020) Simultaneous inhibition of glycolysis and oxidative phosphorylation triggers a multi-fold increase in secretion of exosomes: possible role of 2'3'-cAMP. Sci Rep 10: 6948 32332778 10.1038/s41598-020-63658-5 PMC7181876

[kiac013-B29] Luzarowski M , Vicente R, Kiselev A, Wagner M, Schlossarek D, Erban A, de Souza LP, Childs D, Wojciechowska I, Luzarowska U, et al (2021) Global mapping of protein-metabolite interactions in Saccharomyces cerevisiae reveals that Ser-Leu dipeptide regulates phosphoglycerate kinase activity. Commun Biol 4: 181 33568709 10.1038/s42003-021-01684-3 PMC7876005

[kiac013-B30] Manganiello VC , Degerman E (1999) Cyclic nucleotide phosphodiesterases (PDEs): diverse regulators of cyclic nucleotide signals and inviting molecular targets for novel therapeutic agents. Thromb Haemost 82: 407–411 10605731

[kiac013-B31] Maruri-Lopez I , Figueroa NE, Hernandez-Sanchez IE, Chodasiewicz M (2021) Plant stress granules: trends and beyond. Front Plant Sci 12: 722643 34434210 10.3389/fpls.2021.722643 PMC8381727

[kiac013-B32] Mi H , Huang X, Muruganujan A, Tang H, Mills C, Kang D, Thomas PD (2017) PANTHER version 11: expanded annotation data from Gene Ontology and Reactome pathways, and data analysis tool enhancements. Nucleic Acids Res 45: D183–D189 27899595 10.1093/nar/gkw1138 PMC5210595

[kiac013-B33] Mitreiter S , Gigolashvili T (2021) Regulation of glucosinolate biosynthesis. J Exp Bot 72: 70–91 33313802 10.1093/jxb/eraa479

[kiac013-B34] Moreno JC , Martinez-Jaime S, Kosmacz M, Sokolowska EM, Schulz P, Fischer A, Luzarowska U, Havaux M, Skirycz A (2021) A multi-OMICs approach sheds light on the higher yield phenotype and enhanced abiotic stress tolerance in tobacco lines expressing the carrot lycopene β-cyclase1 gene. Front Plant Sci 12: 624365 33613605 10.3389/fpls.2021.624365 PMC7893089

[kiac013-B35] Moreno JC , Rojas BE, Vicente R, Gorka M, Matz T, Chodasiewicz M, Peralta-Ariza JS, Zhang Y, Alseekh S, Childs D, et al (2021) Tyr-Asp inhibition of glyceraldehyde 3-phosphate dehydrogenase affects plant redox metabolism. EMBO J**:** e106800 34156108 10.15252/embj.2020106800 PMC8327957

[kiac013-B36] Murashige T , Skoog F (1962) A revised medium for rapid growth and bio assays with tobacco tissue cultures. Physiol Plant 15: 473–497

[kiac013-B37] Pabst M , Grass J, Fischl R, Leonard R, Jin C, Hinterkorner G, Borth N, Altmann F (2010) Nucleotide and nucleotide sugar analysis by liquid chromatography-electrospray ionization-mass spectrometry on surface-conditioned porous graphitic carbon. Anal Chem 82: 9782–9788 21043458 10.1021/ac101975k PMC2995335

[kiac013-B38] Perez-Riverol Y , Xu QW, Wang R, Uszkoreit J, Griss J, Sanchez A, Reisinger F, Csordas A, Ternent T, Del-Toro N, et al (2016) PRIDE inspector toolsuite: moving toward a universal visualization tool for proteomics data standard formats and quality assessment of proteomexchange datasets. Mol Cell Proteomics 15: 305–317 26545397 10.1074/mcp.O115.050229 PMC4762524

[kiac013-B39] Rappsilber J , Ishihama Y, Mann M (2003) Stop and go extraction tips for matrix-assisted laser desorption/ionization, nanoelectrospray, and LC/MS sample pretreatment in proteomics. Anal Chem 75: 663–670 12585499 10.1021/ac026117i

[kiac013-B40] Ren J , Mi Z, Stewart NA, Jackson EK (2009) Identification and quantification of 2',3'-cAMP release by the kidney. J Pharmacol Exp Ther 328: 855–865 19033554 10.1124/jpet.108.146712 PMC2646794

[kiac013-B41] Robison GA , Butcher RW, Oye I, Morgan HE, Sutherland EW (1965) The effect of epinephrine on adenosine 3', 5'-phosphate levels in the isolated perfused rat heart. Mol Pharmacol 1: 168–177 5835697

[kiac013-B56] **Salem M, Bernach M, Bajdzienko K, Giavalisco P** (2017) A Simple Fractionated Extraction Method for the Comprehensive Analysis of Metabolites, Lipids, and Proteins from a Single Sample. J Vis Exp 10.3791/55802 PMC5608179 28605387

[kiac013-B42] Sorenson R , Bailey-Serres J (2014) Selective mRNA sequestration by OLIGOURIDYLATE-BINDING PROTEIN 1 contributes to translational control during hypoxia in Arabidopsis. Proc Natl Acad Sci USA 111: 2373–2378 24469793 10.1073/pnas.1314851111 PMC3926019

[kiac013-B43] Steffens A , Jaegle B, Tresch A, Hulskamp M, Jakoby M (2014) Processing-body movement in Arabidopsis depends on an interaction between myosins and DECAPPING PROTEIN1. Plant Physiol 164: 1879–1892 24525673 10.1104/pp.113.233031 PMC3982750

[kiac013-B44] Szklarczyk D , Morris JH, Cook H, Kuhn M, Wyder S, Simonovic M, Santos A, Doncheva NT, Roth A, Bork P, et al (2017) The STRING database in 2017: quality-controlled protein-protein association networks, made broadly accessible. Nucleic Acids Res 45: D362–D368 27924014 10.1093/nar/gkw937 PMC5210637

[kiac013-B45] Tanz SK , Castleden I, Hooper CM, Vacher M, Small I, Millar HA (2013) SUBA3: a database for integrating experimentation and prediction to define the SUBcellular location of proteins in Arabidopsis. Nucleic Acids Res 41: D1185–D1191 23180787 10.1093/nar/gks1151 PMC3531127

[kiac013-B46] Thirumalaikumar VP , Wagner M, Balazadeh S, Skirycz A (2020) Autophagy is responsible for the accumulation of proteogenic dipeptides in response to heat stress in Arabidopsis thaliana. FEBS J 288: 281–292 32301545 10.1111/febs.15336

[kiac013-B47] Thompson JE , Venegas FD, Raines RT (1994) Energetics of catalysis by ribonucleases: fate of the 2',3'-cyclic phosphodiester intermediate. Biochemistry 33: 7408–7414 8003506 10.1021/bi00189a047

[kiac013-B48] Usadel B , Poree F, Nagel A, Lohse M, Czedik-Eysenberg A, Stitt M (2009) A guide to using MapMan to visualize and compare Omics data in plants: a case study in the crop species, Maize. Plant Cell Environ 32: 1211–1229 19389052 10.1111/j.1365-3040.2009.01978.x

[kiac013-B49] Van Damme T , Blancquaert D, Couturon P, Van Der Straeten D, Sandra P, Lynen F (2014) Wounding stress causes rapid increase in concentration of the naturally occurring 2',3'-isomers of cyclic guanosine- and cyclic adenosine monophosphate (cGMP and cAMP) in plant tissues. Phytochemistry 103: 59–66 24735826 10.1016/j.phytochem.2014.03.013

[kiac013-B50] Verrier JD , Jackson TC, Bansal R, Kochanek PM, Puccio AM, Okonkwo DO, Jackson EK (2012) The brain in vivo expresses the 2',3'-cAMP-adenosine pathway. J Neurochem 122: 115–125 22360621 10.1111/j.1471-4159.2012.07705.x PMC3371318

[kiac013-B51] Wen CL , Cheng Q, Zhao L, Mao A, Yang J, Yu S, Weng Y, Xu Y (2016) Identification and characterisation of Dof transcription factors in the cucumber genome. Sci Rep 6: 23072 26979661 10.1038/srep23072 PMC4793291

[kiac013-B52] Wu S , Tohge T, Cuadros-Inostroza A, Tong H, Tenenboim H, Kooke R, Meret M, Keurentjes JB, Nikoloski Z, Fernie AR, et al (2018) Mapping the Arabidopsis metabolic landscape by untargeted metabolomics at different environmental conditions. Mol Plant 11: 118–134 28866081 10.1016/j.molp.2017.08.012

[kiac013-B53] Xu J , Chua NH (2011) Processing bodies and plant development. Curr Opin Plant Biol 14: 88–93 21075046 10.1016/j.pbi.2010.10.003 PMC3042721

[kiac013-B54] Yao Y , Cui X, Al-Ramahi I, Sun X, Li B, Hou J, Difiglia M, Palacino J, Wu ZY, Ma L, et al (2015) A striatal-enriched intronic GPCR modulates huntingtin levels and toxicity. eLife 4: e05449 10.7554/eLife.05449 PMC4372774 25738228

[kiac013-B57] **Zühlke BM, Sokolowska EM, Luzarowski M, Schlossarek D, Chodasiewicz M, Leniak E, Skirycz A, Nikoloski Z** (2021) SLIMP: Supervised learning of metabolite-protein interactions from cofractionation mass spectrometry data. biorxiv 10.1101/2021.06.16.448636

