# Supplementary material for: 2′,3′-cAMP treatment mimics the stress molecular response in Arabidopsis thaliana
Source: Plant Physiol. 2022 Jan 19;188(4):1966–78. doi: 10.1093/plphys/kiac013 (PMC8968299; doi:10.1093/plphys/kiac013)
Supplement: kiac013_Supplementary_Data [file kiac013_supplementary_data.zip › PP2021RR01251DR2_Supplemental_Figure_1.pdf]

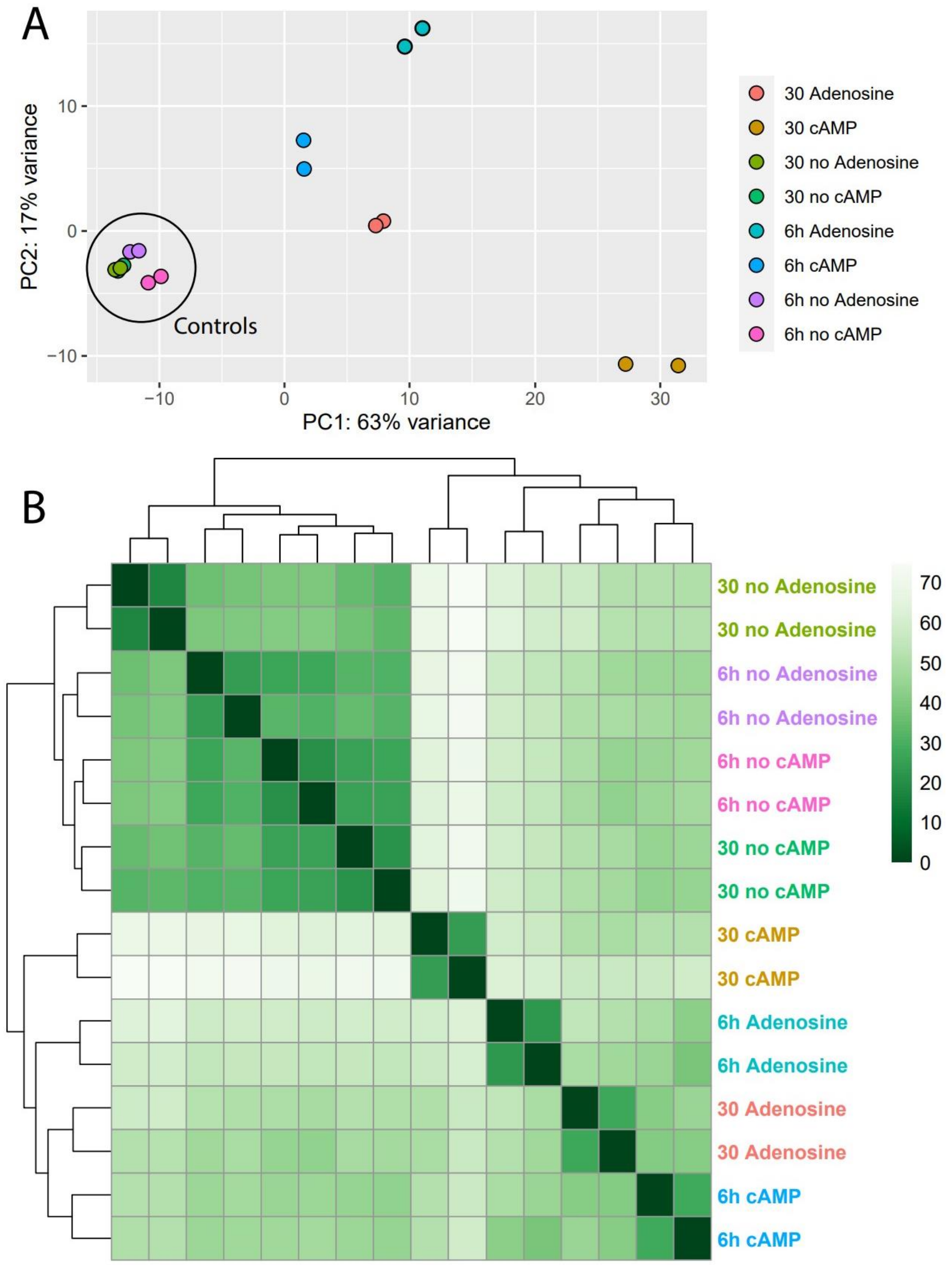

Supplemental Figure S1. Clustering of transcriptomics profiles between replicates of treated and untreated seedlings at two time points. 30 corresponds to 30 min. **A.** PCA representing sample variations. **B.** Sample distance matrix demonstrating reproducibility between replicates.
