# Supplementary material for: 2′,3′-cAMP treatment mimics the stress molecular response in Arabidopsis thaliana
Source: Plant Physiol. 2022 Jan 19;188(4):1966–78. doi: 10.1093/plphys/kiac013 (PMC8968299; doi:10.1093/plphys/kiac013)
Supplement: kiac013_Supplementary_Data [file kiac013_supplementary_data.zip › PP2021RR01251DR2_Supplemental_Figure_2.pdf]

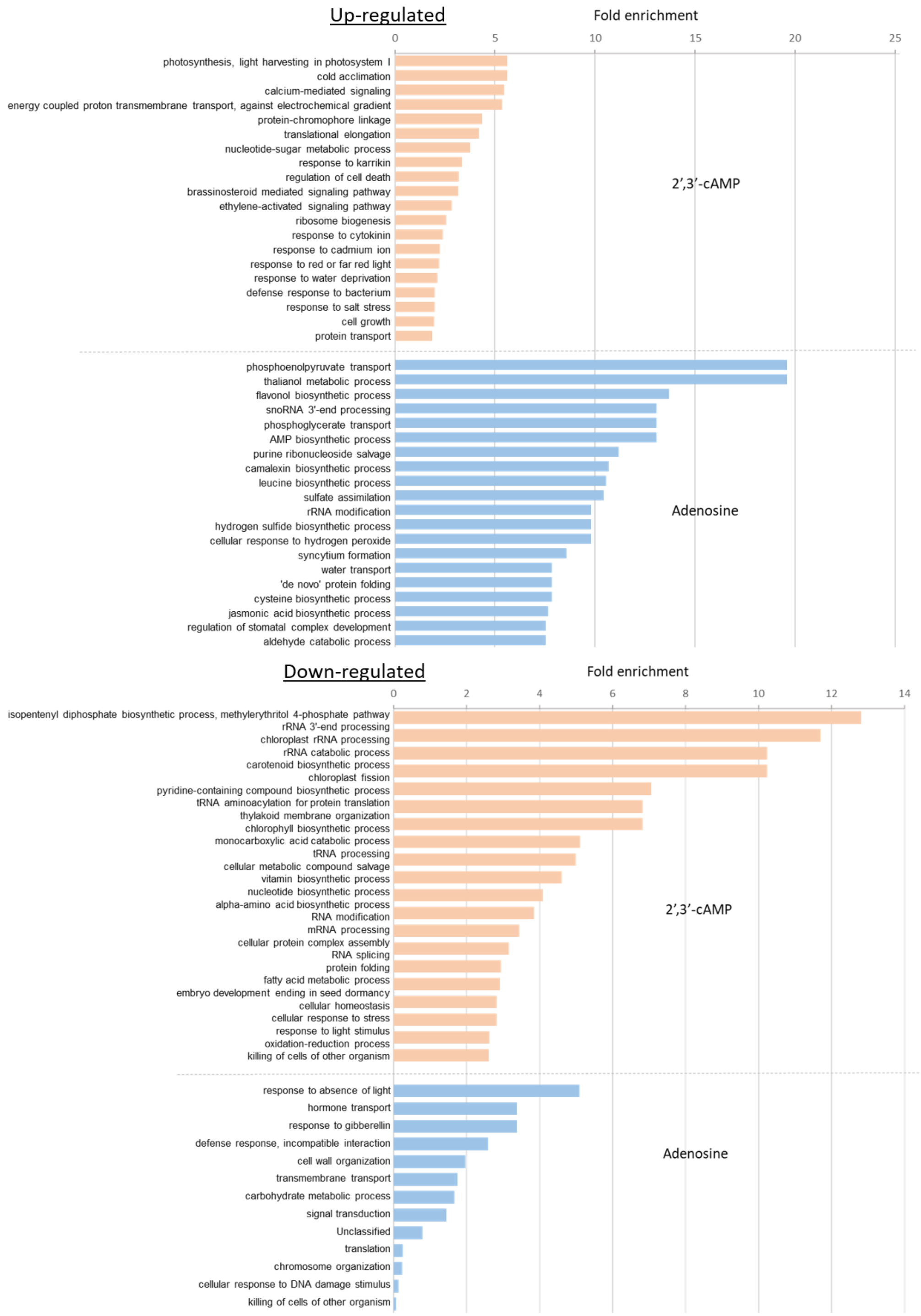

Supplemental Figure S2. Overrepresentation of the biological process in a set of upregulated and downregulated specific genes in 2’,3’-cAMP (orange bars) and adenosine experiments (blue bars). Overrepresentation is shown as a significant fold enrichment based on the PANTHER overrepresentation test (Mi et al., 2017).
