# Supplementary material for: 2′,3′-cAMP treatment mimics the stress molecular response in Arabidopsis thaliana
Source: Plant Physiol. 2022 Jan 19;188(4):1966–78. doi: 10.1093/plphys/kiac013 (PMC8968299; doi:10.1093/plphys/kiac013)
Supplement: kiac013_Supplementary_Data [file kiac013_supplementary_data.zip › PP2021RR01251DR2_Supplemental_Figure_3.pdf]

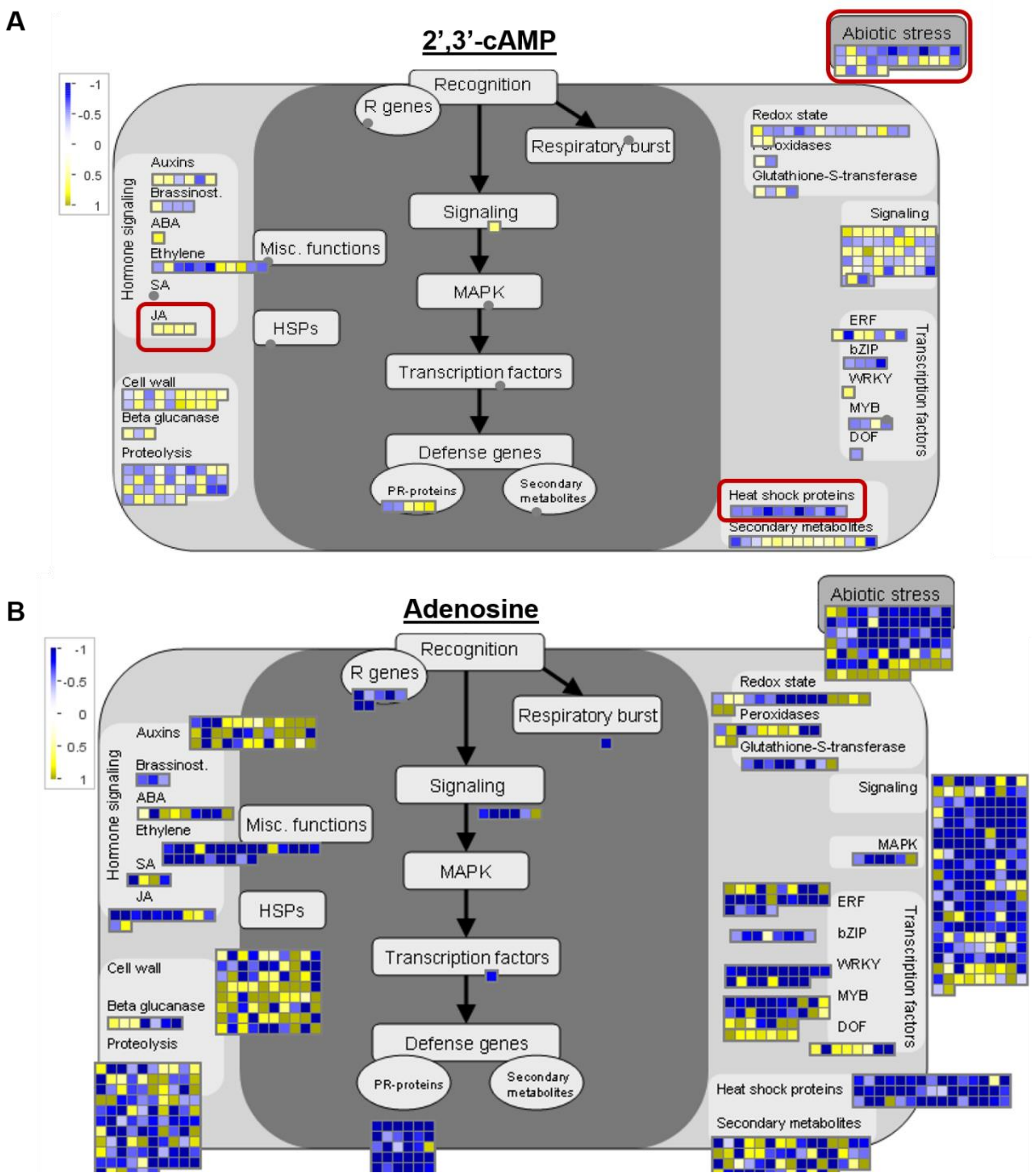

Supplemental Figure S3. MapMan representation of transcriptional perturbations in 30 min time-points for Br-2',3'-cAMP (A) and Br-adenosine treatments (B). Schematic of different processes generated in MapMan depicting hormonal, signaling, and stress response perturbations. Significantly affected DEGs were used for each treatment. The red square represents the most affected processes/components of the biological processes. Scale represents Fold Change. ABA-Absisic Acid, SA-Salicylic Acid, JA- Jasmonic Acid, ERF-Ethylene responsive factors, bZIP-

Basic Leucine Zipper Transcription Factor, WRKY- WRKY-domain containing Transcription Factor, MYB- Myb proto-oncogene Transcription Factor, DOF- DNA-binding One Zinc Finger, HSP- Heat Shock Proteins, MAPK- Mitogen-activated protein kinase.
