# Supplementary material for: 2′,3′-cAMP treatment mimics the stress molecular response in Arabidopsis thaliana
Source: Plant Physiol. 2022 Jan 19;188(4):1966–78. doi: 10.1093/plphys/kiac013 (PMC8968299; doi:10.1093/plphys/kiac013)
Supplement: kiac013_Supplementary_Data [file kiac013_supplementary_data.zip › PP2021RR01251DR2_Supplemental_Figure_5.pdf]

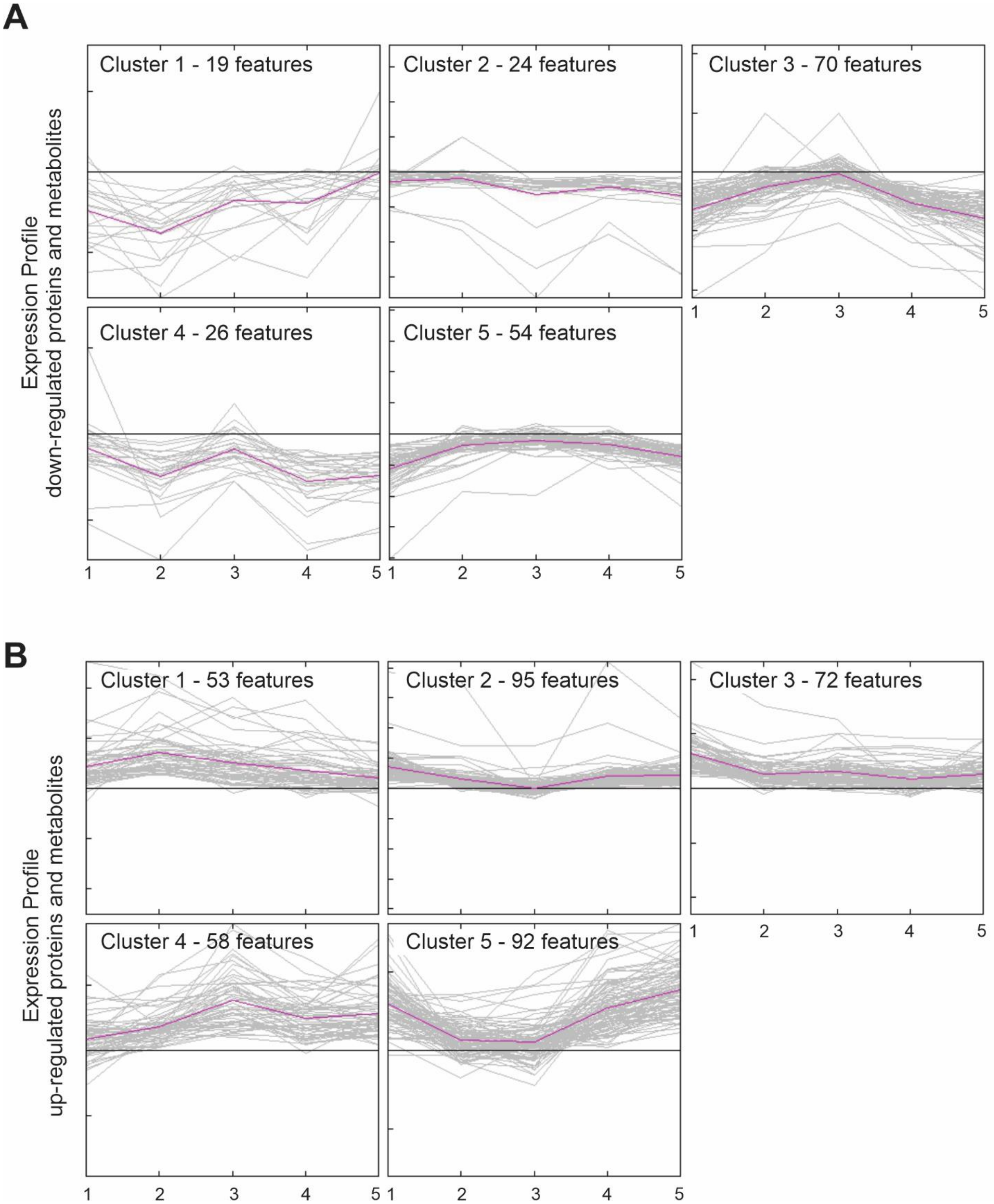

Supplemental Figure S5. Co-expression clustering between proteins and metabolites that are downregulated (**A**) or upregulated by Br-2',3'-cAMP treatment (**B**). Data provided in Supplementary Table 13.
